# Stereotactic Radiofrequency Ablation of Breast Cancer Liver Metastases: Short- and Long-Term Results with Predicting Factors for Survival

**DOI:** 10.1007/s00270-021-02820-6

**Published:** 2021-04-06

**Authors:** Peter Schullian, Edward Johnston, Gregor Laimer, Daniel Putzer, Gernot Eberle, Yannick Scharll, Claudia Ianetti-Hackl, Reto Bale

**Affiliations:** 1 Department of Radiology, Section of Interventional Oncology - Microinvasive Therapy (SIP), Medical University of Innsbruck, Anichstr. 35, Innsbruck, 6020 Austria; 2 Royal Marsden Hospital, 203 Fulham Road, Chelsea, London, SW3 6JJ UK; 3 Visceral, Transplant and Thoracic Surgery, Center of Operative Medicine, Medical University of Innsbruck, Anichstr. 35, Innsbruck, 6020 Austria

**Keywords:** Radiofrequency ablation, Stereotaxy, Breast cancer liver metastasis

## Abstract

**Purpose:**

To evaluate safety, local oncological control, long-term outcome and potential prognostic factors of stereotactic RFA (SRFA) for the treatment of BCLMs.

**Methods:**

Between July 2003 and December 2019, 42 consecutive female patients with median age 54.0 years were treated with SRFA at our institution for 110 BCLMs in 48 ablation sessions. Median tumor size was 3.0 cm (0.8–9.0). Eighteen (42.9%) patients had extrahepatic metastasis at initial SRFA.

**Results:**

Technical success rate was 100%, i.e., all coaxial needles were inserted with appropriate accuracy within 10 mm off plan and 107/110 (92.3%) BCLMs were successfully ablated at initial SRFA. Four Grade 1 (8.3%, 4/48) and one Grade 2 (2.1%, 1/48) complications occurred. No perioperative deaths occurred. Local recurrence developed in 8 of 110 tumors (7.3%). Overall survival (OS) rates of all patients at 1, 3, and 5 years from the date of the first SRFA were 84.1%, 49.3%, and 20.8% with a median OS of 32.3 months. Univariable cox regression analyses revealed age > 60 years and extrahepatic disease (without bone only metastases) as significant predictors of worse OS (*p* = 0.013 and 0.025, respectively). Size and number of metastases, hormone receptor status and time onset did not significantly affect OS after initial SRFA.

**Conclusions:**

SRFA is a safe, minimally invasive treatment option in the management of BCLMs, especially in younger patients without advanced extrahepatic metastasis, including those with large liver tumors.

## Introduction

Breast cancer is one of the most common malignancies in women and is a leading cause of mortality worldwide [1]. Approximately, 20% of breast cancer patients develop metastatic disease [2], with the lungs, liver, bone and brain being the most common sites. Breast cancer liver metastasis (BCLM) confer a poor prognosis of 4–8 months survival [3] and are found in approximately 50% of patients with metastatic disease, where 5–12% of patients have liver only metastases [3]. However, patients with negative resection margins after surgery show 5-year survival rates up to 40% [4].

Advanced breast cancer is primarily treated by systemic hormone therapy and/or chemotherapy, and despite advancements in systemic treatment, median overall survival and 5-year survival rates remain low, at 18–24 months and 27%, respectively [5].

Recent studies suggest that subgroups of breast cancer patients with oligometastatic disease benefit from additional locoregional treatment [6, 7], which is defined by the 3rd ESO–ESMO (European School of Oncology–European Society for Medical Oncology) consensus guidelines as limited metastatic disease with up to five metastases that are potentially amenable for local treatment [8].

Percutaneous thermal ablation methods, such as radiofrequency ablation (RFA) and microwave ablation (MWA) have gained widespread acceptance as a minimally invasive treatment option in the management of primary and metastatic liver tumors [9–12]. Despite several studies showing promising results for RFA in BCLM [13, 14], high-quality evidence is still lacking.

Stereotaxy (derived from the Greek meaning “solid arrangement”), allows the planning of complex trajectories using three-dimensional image datasets with precise transformation into real patients using a Cartesian coordinate system [15]. Furthermore, fusion with previously acquired MR images in case of poor tumor visibility, immediate post-ablation contrast-enhanced CT fusion with the planning CT for reliable assessment of ablation results, allows more complex interventions such as large tumors and those in challenging localizations such as the hepatic dome or caudate lobe.

The purpose of the present study was to evaluate safety, local oncological control, long-term outcome and potential prognostic factors of stereotactic RFA (SRFA) for the treatment of BCLMs.

## Materials and Methods

### Patient Selection

The local institutional review board approved this retrospective single-center study, and all patients included gave their informed consent. Each case was reviewed and the treatment plan was approved by consensus in multidisciplinary tumor advisory board meetings.

One thousand seven consecutive patients were treated by SRFA between July 2003 and December 2019. Twenty-eight patients who underwent SRFA for benign liver tumors were excluded. Forty-two consecutive patients with BCLM were treated in 48 ablation sessions and included in the study. Table 1 shows the baseline characteristics of the study group.

**Table 1:** Patient characteristics of 42 patients with 110 breast cancer liver metastasis undergoing 48 SRFA for local treatment

Patient characteristics	Study Gr.
Age, median years (range)	54.0 (31–82)
Sex (female/male), *n* (%)	42/0 (100/0)
Tumor size, median (range)	3.0 cm (0.8–9.0)
Tumor number at begin, *n* (range)	2 (1–8)
* n* = 1, *n* (%)	16 (38.0)
*n* = 2, *n* (%)	13 (31.0)
*n* ≥ 3, *n* (%)	13 (31.0)
Coaxial needles per session, *n* (range)	9 (3–16)
Time onset of BCLM, median months (range)	30.5 (0–136)
Extrahepatic metastasis before SRFA	18 (42.9)
Bone, *n* (%)	6 (14.3)
Bone and lung *n* (%)	4 (9.5)
Lung, *n* (%)	4 (9.5)
Lymph nodes, *n* (%)	1 (2.4)
Bone and lymph nodes, *n* (%)	3 (7.1)
Treatment before SRFA	
CTX, *n* (%)	19 (45.2)
HT, *n* (%)	2 (4.8)
Combined CTX and HT, *n* (%)	17 (40.5)
Combined CTX, HT and HR, *n* (%)	4 (9.6)
Treatment after SRFA	
CTX, n (%)	18 (42.9)
HT, n (%)	2 (4.8)
Combined CTX and HT, *n* (%)	18 (42.9)

*BCLM* breast cancer liver metastasis, *SRFA* stereotactic radiofrequency ablation, *HR* hepatic resection, *CTX* chemotherapy, *HT* hormone therapy, *cRFA* conventional RFA

Exclusion criteria for SRFA comprised (i) platelet count < 50,000/mm3 (ii) prothrombin activity < 50% and (iii) tumor location close to (< 10 mm) the central bile ducts. Tumor diagnosis was confirmed by multiphasic contrast MRI or CT and inconclusive cases were validated by biopsy.

### SRFA Procedure

**Fig. 1:**
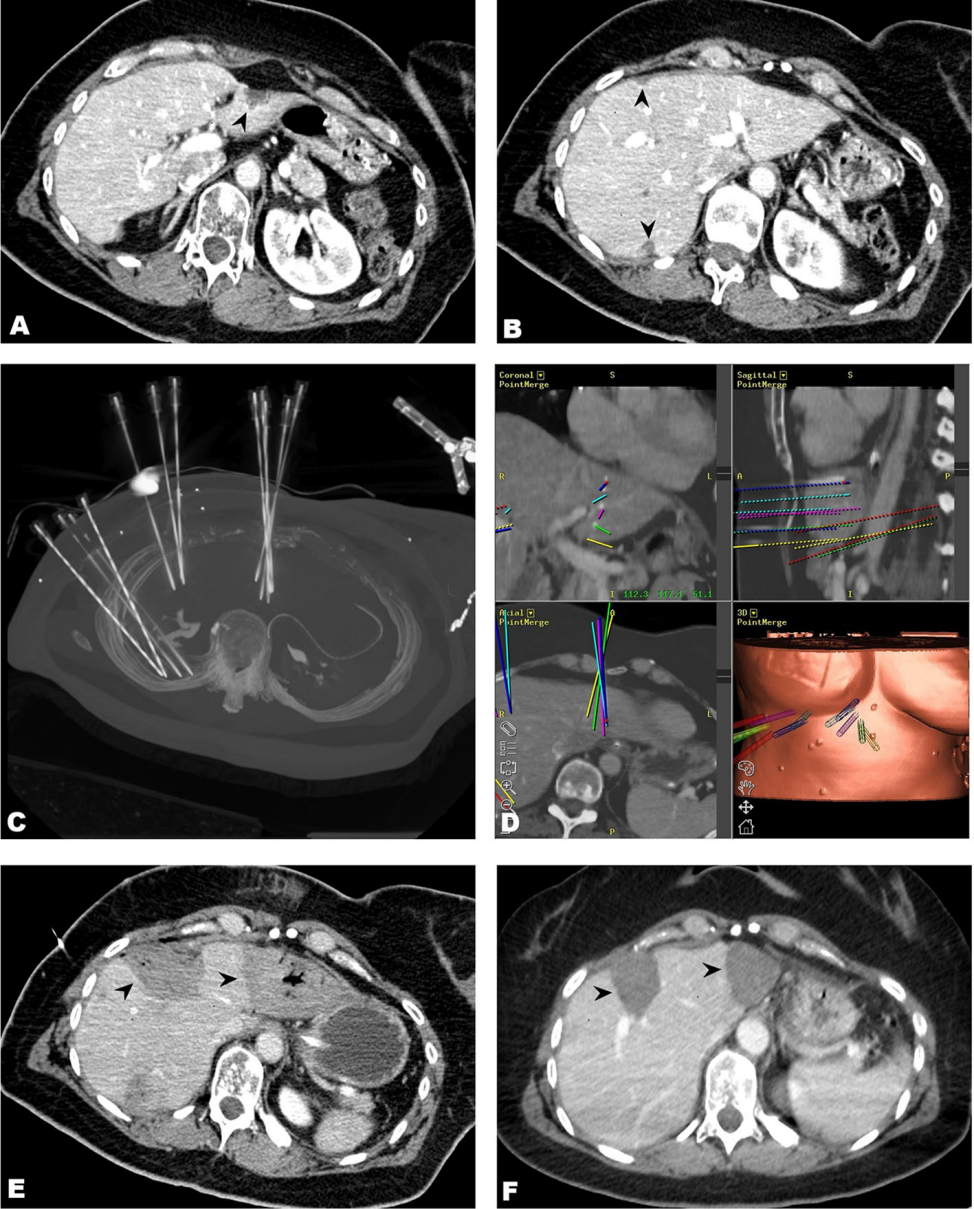
A 50-year-old female with breast cancer liver metastases measuring up to 3.0 cm. **A** Contrast enhanced CT-image of one BCLM (black arrowhead) in segment II/III close to the stomach. **B** Contrast enhanced CT-image showing the other two subcapsular BCLMs in the right lobe (black arrowhead). **C** Maximum Intensity Projection image of the control CT with 12 coaxial needles in place. **D** Fused images of the control CT with the planning CT showing superposition of planned paths with inserted coaxial needles. **E, F** Black arrowheads demonstrating ablation zones on CT images immediately after the procedure and after 36 months later without evidence of local recurrence

The method of SRFA has been reported in detail previously [16–18]. An example SRFA for BCLM is shown in Fig. 1.

### Outcome Measurements

Sufficiently precise coaxial needle placement (deviation from plan < 1 cm at each needle tip) was defined as a technical success. Technical efficacy and local recurrence rate (LR) were determined by contrast-enhanced CT or MR follow-up examinations performed at intervals of 1 month and 3 months after SRFA. Images were evaluated in consensus by two experienced abdominal radiologists (radiologist 1 with 20 years of experience and radiologist 2 with 11 years of experience). Primary technical efficacy rate was evaluated for each tumor, defined as the absence of residual disease on the 1-month follow-up CT. Secondary technical efficacy rate was defined as tumors requiring repeat ablation due to residual tumor. Appearance of new nodules within or immediately adjacent to ablation zones or the original tumor was defined as LR. New nodules distant to the ablation zone and / or the original tumor were defined as distant tumor recurrence. Complications were defined according to the CIRSE Classification System for Complications [19]. Disease-free (DFS) and overall survival (OS) were calculated from the date of initial SRFA until the date of death due to malignancy or other causes (i.e., event), until date of relapse (DFS) or until the last follow-up visit (i.e., censoring).

### Prognostic Factors

Age (> / < 60 years), tumor size (> / ≤ 3 cm), tumor number (> / = 1), tumor distribution (uni-/bilobar), presence of extrahepatic disease, time from primary tumor to BCLM (> / < 24 months) and hormone receptor status were evaluated as potential prognostic factors for OS.

### Statistics

Statistical analysis was performed using IBM-SPSS v24. Data were expressed as total numbers, median and range. Differences between categorical variables were evaluated using the *X*^2^ test and between independent continuous variables using the Mann–Whitney U test. OS and DFS were evaluated using the Kaplan–Meier method. Cox regression was used to analyze potential factors of OS. The variables of interest (*p* < 0.1) identified in the univariable analysis were further analyzed in a multivariable analysis using the cox regression model. *p* < 0.05 was considered statistically significant.

## Results

### Patient Characteristics

In the study group, 42 females with a total of 110 BCLMs had a median age of 54.0 (31.0–82.0). Median tumor size was 3.0 cm (0.8–9.0), and a median of 2 BCLM (1–6) were treated per ablation session (48 sessions in total). At baseline (initial SRFA), 16 (38.0%) patients had a solitary liver metastasis, 13 (31.0%) had two metastases, and 13 (31.0%) patients had three or more liver metastases. BCLMs were synchronous metastases in 7 (16.7%) patients and metachronous in 35 (83.3%). The median time between primary diagnosis and detection of BCLM was 30.5 months (range 0–136 months). Eighteen (42.9) patients had extrahepatic metastasis, with 6 (14.3%) in bone, 4 (9.5%) in bone and lung, 4 (9.5%) in lung, 1 (2.4%) in lymph nodes and 3 (7.1%) in bone and lymph nodes. Before SRFA, 19 (45.2%) patients underwent chemotherapy, 2 (4.8%) hormone therapy, 17 (40.5%) combined treatment of chemotherapy and hormone therapy and 4 (9.6%) a combined treatment of chemotherapy, hormone therapy and hepatic resection. No patient received immunotherapy. After SRFA, 18 (42.9%) patients received chemotherapy, 2 (4.8%) hormone therapy and 18 (42.9%) a combination of hormone and chemotherapy. See Table 1 for details.

During ablation, BCLMs were confirmed histologically in 18 of 42 (42.3%) patients. In one patient the result was inconclusive due to insufficient histological material. Of the remaining 23 patients, BCLMs were confirmed histologically before ablation in 20 patients, and no histological result was available in 3 patients.

### Periprocedural Complications

According to the CIRSE Classification System for Complications, four Grade 1 (8.3%, 4/48) and one Grade 2 (2.1%, 1/48) complications occurred. Four patients developed arterial bleeding from subcapsular liver vessels, managed by transarterial coil embolization in the same anesthetic session. One major pleural effusion required treatment with a chest tube. Median hospital stay after SRFA was 4.5 days, ranging from 2–39 days. No perioperative deaths occurred.

### Local Tumor Control and Distant Tumor Progression

Technical success rate was 100%, i.e., all coaxial needles were inserted with appropriate accuracy within 10 mm off plan. 107/110 liver metastases were successfully ablated at initial SRFA (97.3% primary technical efficacy rate), whereby 1 of 3 tumors were successfully treated in a second session, resulting a secondary technical efficacy rate of 98.2%. Local recurrence (LR) developed in 8 of 110 tumors (7.3%) after a median imaging follow-up of 10.9 months (range 1.4–112 months). Details for insufficient local control are presented in Table 2. An overview of success rates is provided in Table 3.

**Table 2:** Details of local treatment failures after SRFA

ID	Age	Size (cm)	Needles	Ablation time (min)	Segment	Location properties	Outcome
1	51	3.0	5	48	IVb	gb	iA
2	44	7.0	8	80	VI, VII	v	iA
3	45	3.0	5	40	IVa	sc	iA
4	63	6.5	9	51	V, VI	–	LR
5	50	5.5	8	54	III, IVb	v, sc,	LR
6	70	2.0	2	16	II	sp	LR
7	70	2.4	2	16	IVb	sc	LR
8	70	1.8	2	12	IVb	–	LR
9	70	1.0	1	12	II	v	LR
10	45	2.0	3	32	III	o	LR
11	51	6.2	13	70	VI, VII	sc	LR

*SRFA* stereotactic radiofrequency ablation, *v* close to major vessel, *sc* subcapsular, *sp* subphrenic, *o* close to organ, *gb* close to gallbladder, *iA* incomplete ablation, *LR* local recurrence

**Table 3:** Tumor-based therapy success rates

Rate	
Technical success, *n* (%)	110/110 (100)
Primary technical efficacy, *n* (%)	107/110 (97.3)
Secondary technical efficacy, *n* (%)	108/110 (98.2)
Local recurrence, *n* (%)	8/110 (7.3)

During follow-up, 19/42 (45.2%) patients developed disease-progression, with 12 (28.6%) developing multiple new liver metastases, 3 (7.1%) developing extrahepatic metastases and 8 patients (19.0%) both multiple new liver metastases and extrahepatic metastases.

There was no significant difference in LR for lesions > 3 cm and < 3 cm with LR rates of 11.5% (3/26) and 6.0% (5/84), respectively (*p* = 0.338). A tumor location close to organs (LR 11.1%, 1/9 vs. all other locations with 6.9%, 7/101; *p* = 0.644), close to the liver capsule (LR 8.3%, 3/36 vs. all other locations with 6.8%, 5/74; *p* = 0.765), close to the diaphragm (LR 10.0%, 1/10 vs. all other locations with 7.0%, 7/100; *p* = 0.728) or to large vessels (LR 11.1%, 2/18 vs. all other locations with 6.5%, 6/92; *p* = 0.493) did not significantly affect LR.

### Overall and Disease-Free Survival (Figure 2 and Figure 3)

**Fig. 2:**
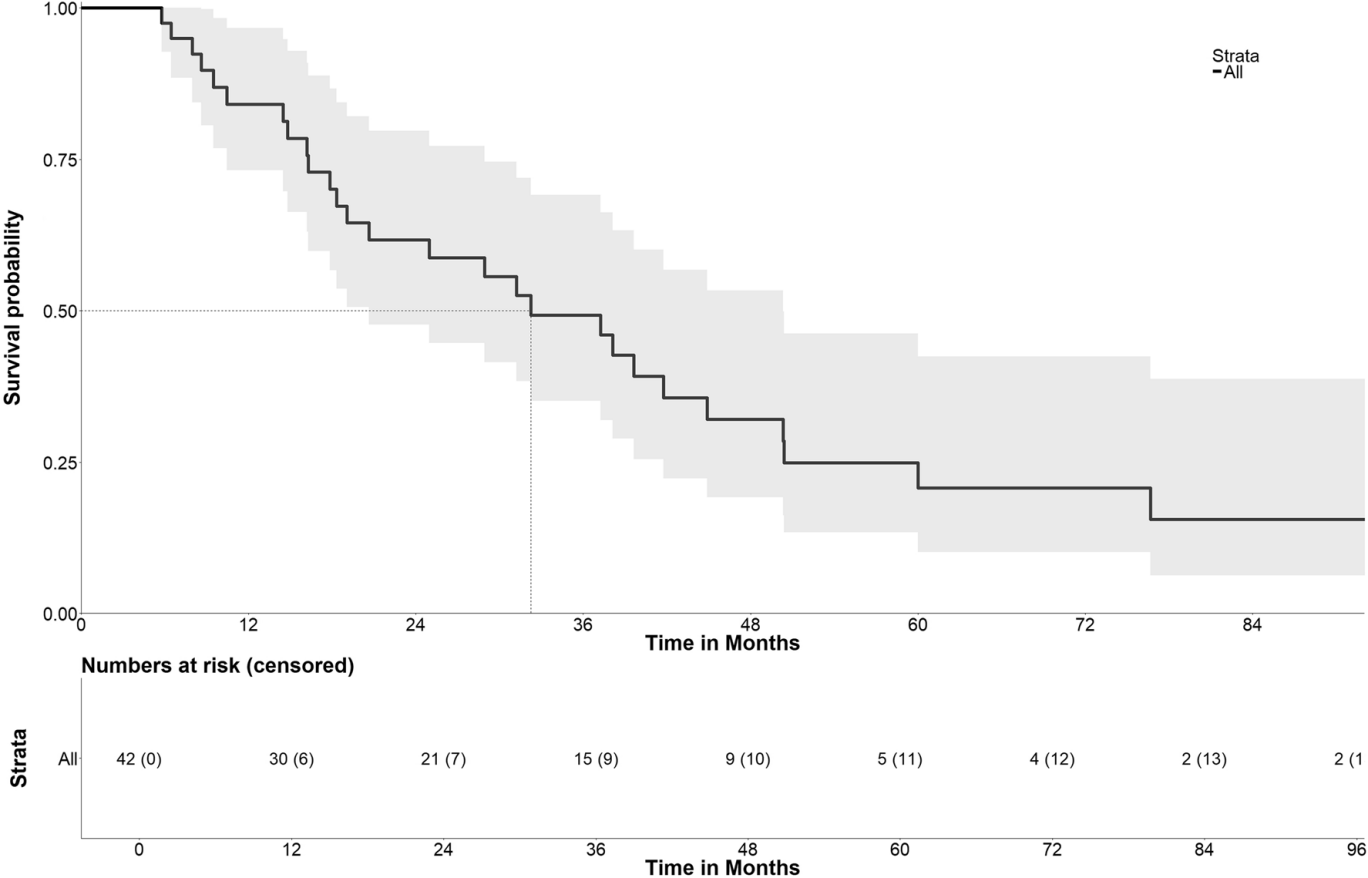
Overall survival after initial SRFA

**Fig. 3:**
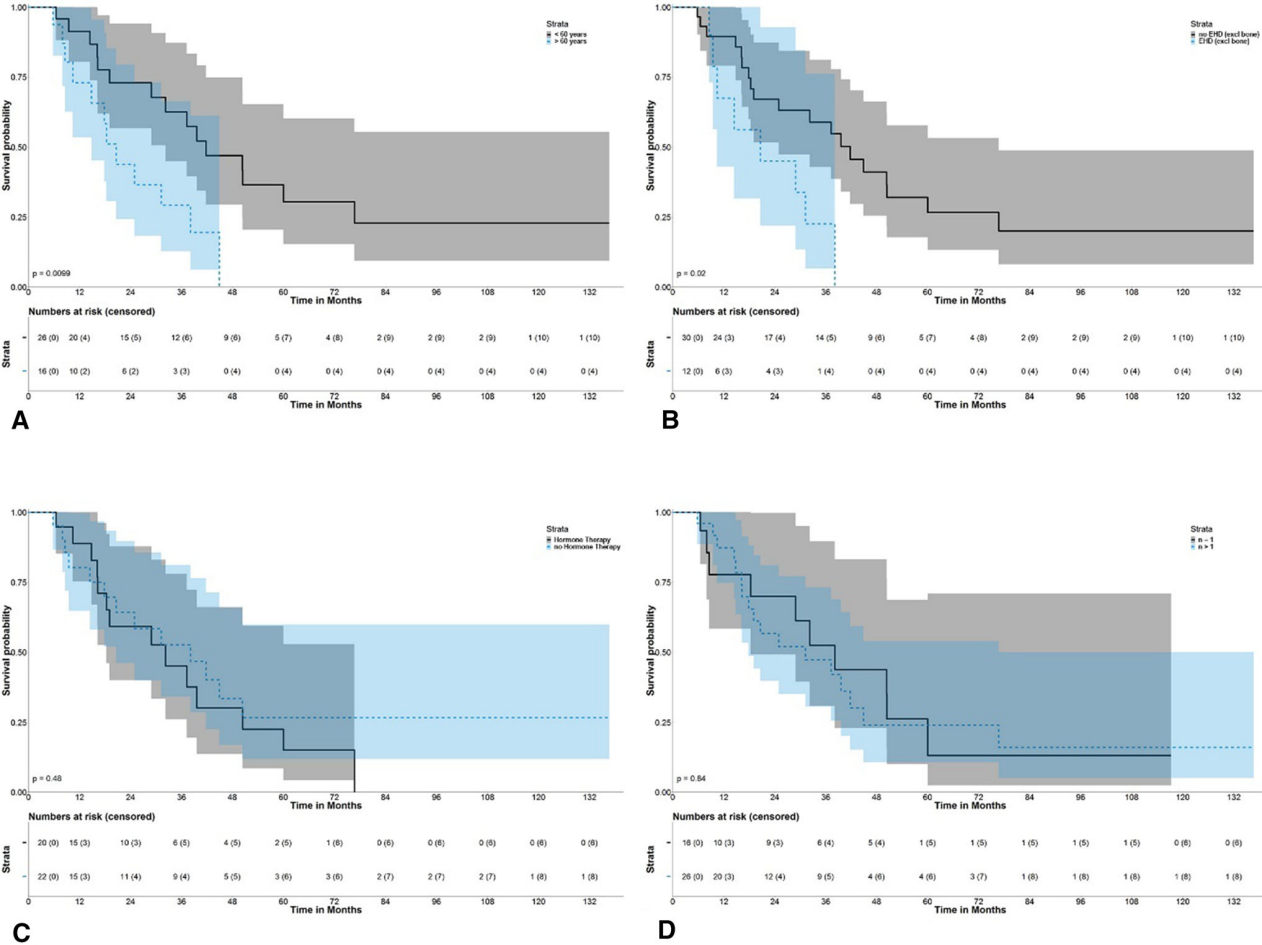
Overall survival after initial SRFA grouped by age (**A**), extrahepatic disease excluding bone only metastasis (**B**), hormone therapy (**C**) and number of tumors (**D**)

Overall survival (OS) rates of all patients at 1, 3, and 5 years from the date of the first SRFA were 84.1%, 49.3%, and 20.8% with a median OS of 32.3 months (95% CI 20.6–50.3). The corresponding OS rates at 1, 3, and 5 years from the date of tumor diagnosis were 97.6%, 87.8%, and 72.3% with a median OS of 93.2 months (95% CI 65.6–120.8) and 92.5%, 62.6%, and 35.9% with a median OS of 48.2 months (95% CI 39.9–56.5) from the date of liver metastasis diagnosis.

Univariable cox regression analyses revealed age > 60 years (*p* = 0.013, HR 2.3 CI 0.9–6.0) and extrahepatic disease (excluding patients with bone only metastasis; *p* = 0.025, HR 1.8 CI 0.7–5.1) as significant predictors of worse OS. Median OS in BCLM patients without extrahepatic disease at initial SRFA was 41.8 months with OS rates at 1, 3, and 5 years of 89%, 59% and 27%. By comparison, BCLM patients younger than 60 years showed better survival with a median OS 41.8 and OS rates at 1, 3, and 5 years of 92%, 63% and 30%. Size and number of BCLM, hormone receptor status and time onset of BCLM did not significantly affect OS after initial SRFA. Full details are provided in Table 4.

**Table 4:** Cox regression analyses of factors affecting overall survival (OS) after initial SRFA

Variables	OS rates (%)			OS_±_	Univariate analyses *p* value	Multivariable analysis		
	1 yr	3 yr	5 yr			*p* value	Hazard ratio	95% CI
Age					0.013*	0.090	2.301	0.878–6.031
> 60 yr old	73	28	–	20.6				
< 60 yr old	92	63	30	41.8				
Tumor size					0.171			
> 3 cm	92	49	06	32.2				
≤ 3 cm	70	51	51	76.7				
Tumor number					0.838			
> 1	88	46	25	31.2				
1	79	55	15	38.1				
Tumor distribution					0.423			
Unilobar	81	51	14	37.3				
bilobar	92	46	36	31.2				
Extrahepatic disease					0.208			
Yes	82	37	13	28.9				
No	87	57	25	39.6				
Extrahepatic disease (excluding bone only)					0.025*	0.246	1.835	0.658–5.120
Yes	68	23	0	20.6				
No	89	59	27	41.8				
Time to BCLM					0.129			
< 24 months	75	68	37	50.4				
> 24 months	91	35	8	29.9				
Hormone receptor					0.479			
Positive	80	52	26	38.1				
Negative	89	46	15	32.3				

*p * < 0.05 is considered as statistical significance (bold and asterisk)

*OS* overall survival, ± median (months), *yr* year,

Multivariable cox regression analyses showed no independent prognostic factors for OS. Disease-free survival (DFS) for all patients at 1, 3, and 5 years from the date of the first SRFA were 45.3%, 22.3%, and 15.9% with a median OS of 10.5 months (95% CI 6.8–25.0).

## Discussion

The results of the present study suggest that patients with breast cancer liver metastases benefit from stereotactic radiofrequency ablation (SRFA). More specifically, we found a median OS of 32.2 months from the date of BCLM treatment, which is considerably higher than no treatment, at 3–15 months [20, 21].

In contrast to the substantial evidence behind resection for colorectal liver metastasis, data regarding BCLM are limited. In a systematic review of 33 papers, Fairhurst et al. [22] reported a median OS of 35.1 months and a 5-year survival rate of 33% after BCLM resection, which is closely aligned with the data of the present study. Newer studies such as Ruiz et al. [23] reported better OS when combining resection with systemic treatment, with a median OS of up to 82 months for liver confined metastases. Regarding survival after conventional RFA in patients with BCLM, several authors showed promising results. The reported median OS rates of several studies range from 26–29.9 months with OS rates at 1, 3 and 5 year of 68–90%, 25–44% and 11–27% [14, 24, 25]. These studies included mainly small tumors up to 3 cm due to the limitations of conventional targeting techniques. Despite the fact that the majority (62%) of patients in the present study had multiple liver metastases with a median size of 3 cm (up to 9 cm), our median OS of 32.2 months, with 1-, 3- and 5-year OS rates of 84.1%, 49.3% and 20.8% compare favorably to the literature regarding conventional RFA.

Patient selection, especially in the context of advanced breast cancer patients, is a crucial step towards improving outcomes, which could be improved by identifying prognostic factors associated with better survival. Positive hormone receptor status has been reported as a positive predictive factor for survival after hepatic resection for BCLM [3, 26, 27]. However, we found no evidence for such an association (*p* = 0.479), maybe due to the selected study cohort. In line with our results, Jakobs et al. [28] reported that hormone receptor status did not significantly affect survival after conventional RFA for BCLM. Late onset of BCLM has also been proposed as a predictor of survival by He et al. [29] and Hoffman et al. [30] in the surgical literature, although this is again distinct from findings in the RFA literature, including this study. One possible explanation for this difference could be due to the different selection criteria for hepatic resection vs. RFA. Another important reported prognostic factor of survival is the presence of extrahepatic disease at initial therapy, where we found extrahepatic metastases (excluding patients with isolated bone metastases) might be significantly associated with reduced survival (*p* = 0.025), which is an observation supported by Jakobs et al. [28] after conventional RFA.

Several studies [14, 24, 31] have also shown significantly reduced survival in patients with BLCMs > 2.5 cm after RFA. Whilst larger tumors were associated with worse survival in our study, this did not reach statistical significance (*p* = 0.171, Table 4). In addition, Cox regression analysis revealed age < 60 years was a positive predictor of survival, which is in line with the results of Dittmar et al. [32] following hepatic resection for BCLM.

Owing to the advantages of our setup, the technical success rate, i.e., accurate needle placement (deviation ≤ 10 mm from the plan) was 100%. The measurement of the safety margin to determine success represents another important outcome measure, which we have not (yet) used in the present study. However, the importance of an adequate safety margin for local tumor control in patients with colorectal liver metastases and hepatocellular carcinoma has been addressed by our study group recently [33, 34].

Insufficient local control remains a major drawback of conventional RFA, especially for larger tumors with reported local recurrence rates between 14 and 50% [14, 35]. As such, to achieve complete ablation in larger tumors, multiple overlapping ablation volumes are required [36], although the resulting increase in complexity using multiple needles and pathways is very difficult to achieve with conventional techniques. We have therefore developed a technique which uses sophisticated 3D planning, translation of the plan to the patient using a frameless stereotactic navigation system paired with a neurosurgical aiming device and ablation zone evaluation using image fusion. We recently published a study of 97 patients undergoing SRFA for HCC prior to liver transplantation, and demonstrated complete pathological response in 183 of 188 nodules (97.3%), and in 50 of 52 nodules > 3 cm (96.2%) [37]. Besides RFA, microwave ablation (MWA) is an important ablative strategy which has a higher, and faster thermal energy transfer [38], which allows for larger ablation zones. However, studies regarding MWA for BCLM are sparse, with small patient numbers and ultimately inconclusive results.

Whilst reported LR rates after conventional RFA for BCLM are between 11.6 and 25% [14, 24, 25], the majority of lesions are < 3 cm. In comparison, our reported LR rate of 7.3% compares favorably to these results, given a median tumor size of 3 cm, which we attribute to our scrupulous technique, with the aim of achieving a sufficient ablation margin of at least 5 mm. This is achieved through the use of a sophisticated aiming device with precise needle placement and planning software which also allows fusion with previously acquired MR images in case of poor target visibility. Immediate post-ablation contrast-enhanced CT fusion with planning CT allows rapid and reliable assessment of ablation results with the option of repeat ablation in the same session. This standardized approach to ablation means tumors can be reliably treated without limitation in size [39] (the largest lesion treated in this cohort was 9 cm) and number [40]. Finally, in our experience, SRFA is easier to learn than conventional techniques, because important workflows can be trained under laboratory conditions, and the learning curve can be ascended before use in patients.

The mortality and complication rate (Grade 1 & 2) in our study were 0% and 10.4% (5/110), respectively, which is considerably higher than reported complication rates for conventional ablation, which range from of 0 to 1.1% [14, 24, 41]. The explanation for this is very likely to be due to the higher complexity of interventions, whereby tumors are often large and/or multiple. However, 4 out of 5 of the complications were successfully treated in the same anesthetic session and did not change the postoperative course. Nevertheless, our results compare favorably to hepatic resection for BCLM, which confers 0–5.9% mortality and 15% major morbidity [22].

## Limitations

The limitations of this study include its retrospective design and a relatively small sample size. This small sample size reduces the accuracy of the subgroup analyses in particular. Use of additional therapies, such as chemotherapy and/or hormone therapy after SRFA should also impact the overall clinical outcome. Furthermore, comparison with previous studies is limited as stereotactic navigation systems were not employed in prior reports.

In conclusion, SRFA is a safe, minimally invasive treatment option in the management of BCLMs for selected patients who might benefit from local treatment, with similar survival rates to hepatic resection.

## Abbreviations

BCLM: Breast cancer liver metastasis
DFS: Disease-free survival
LR: Local recurrence
OS: Overall survival
SRFA: Stereotactic radiofrequency ablation
